# Human Peripheral Clocks: Applications for Studying Circadian Phenotypes in Physiology and Pathophysiology

**DOI:** 10.3389/fneur.2015.00095

**Published:** 2015-05-13

**Authors:** Camille Saini, Steven A. Brown, Charna Dibner

**Affiliations:** ^1^Department of Medical Specialties, Faculty of Medicine, University of Geneva, Geneva, Switzerland; ^2^Institute of Pharmacology and Toxicology, University of Zürich, Zürich, Switzerland

**Keywords:** circadian clock, human chronotype, human primary cells, skin fibroblasts, bioluminescence

## Abstract

Most light-sensitive organisms on earth have acquired an internal system of circadian clocks allowing the anticipation of light or darkness. In humans, the circadian system governs nearly all aspects of physiology and behavior. Circadian phenotypes, including chronotype, vary dramatically among individuals and over individual lifespan. Recent studies have revealed that the characteristics of human skin fibroblast clocks correlate with donor chronotype. Given the complexity of circadian phenotype assessment in humans, the opportunity to study oscillator properties by using cultured primary cells has the potential to uncover molecular details difficult to assess directly in humans. Since altered properties of the circadian oscillator have been associated with many diseases including metabolic disorders and cancer, clock characteristics assessed in additional primary cell types using similar technologies might represent an important tool for exploring the connection between chronotype and disease, and for diagnostic purposes. Here, we review implications of this approach for gathering insights into human circadian rhythms and their function in health and disease.

## Introduction: The Web of Body Circadian Clocks

During evolution, most light-sensitive organisms living on earth, including mammals, adapted to daily and seasonal variations of luminosity and temperature resulting from the earth’s movement. An internal timing system allowing measuring time, anticipating environmental daily changes, and tuning physiology and behavior to these variations has therefore been developed. Under constant conditions, without any light and temperature variations, this internal timing system keeps “free-running.” It drives cyclic physiology and behavior with a period of approximately, but not precisely, 24 h. Hence, this anticipatory internal timing system was named the “circadian clock,” from the Latin “*circa diem*” meaning “about a day,” reflecting this timekeeper’s need to be adjusted on a daily basis by external time cues. The first recognition of this phenomenon was provided already in 1729 by Jean-Jacques d’Ortous de Mairan, who observed that the circadian movement of *Mimosa pudica* leaves was preserved in constant darkness (1). However, it took more than two centuries until the first clues about circadian clock molecular cogwheels started to appear. In humans, the intrinsic period of the circadian pacemaker (τ) probably averages slightly longer than 24 h (2–5). Molecular circadian clocks are present in virtually all body cells. This complex body oscillator network keeps its synchrony owing to a small group of pacemakers located in neurons of the hypothalamic suprachiasmatic nuclei (SCN), the central clock, which is synchronized every day by retinal signals emanating from light. In turn, the central clock uses diverse and not entirely unraveled pathways to reset the phase of peripheral (or slave) oscillators (6, 7). The connection between SCN and peripheral clocks proceeds via a plethora of neural and endocrine pathways, or indirectly through the control of the rest/activity cycle, the resulting fasting/feeding and metabolic cycles, as well as through daily oscillations of body temperature. Light signals represent the most important synchronization cue, or *Zeitgeber*. In addition, a variety of external stimuli such as temperature, nutrient availability, or social interactions may contribute to phase resetting of the circadian clock (8). The circadian clock drives virtually all biological processes occurring in the organism in order to synchronize them to geophysical time. The major purpose of this adaptation is the orchestration of key metabolic processes, including food processing (anabolism, catabolism, detoxification), in anticipation of corresponding feeding/fasting and activity/rest episodes and therefore in the most efficient manner in terms of energy balance (9). The current molecular model for the generation of circadian oscillations is based on interlocked negative feedback loops of clock gene expression and protein translation. In humans, similarly to other mammals, the major loop comprises two PAS-domain helix-loop-helix transcriptional activators BMAL1 and CLOCK, which form a heterodimer that activates the transcription of the negative core-clock limb actors. The negative actors, members of the PER and CRY protein families, accumulate and negatively feed back on their own transcription [for detailed model, see Ref. (10)]. Beyond this transcription–translation loop, posttranslational events such as the control of protein phosphorylation, sumoylation, acetylation, O-GlcNAcylation, degradation, and nuclear entry, as well as extensive chromatin modification, contribute critically to the generation of daily oscillations in clock gene products [(11) and references therein].

## A Link between Human Chronotype, Chronic Circadian Misalignment, and Disease

Chronotype reflects the tendency of each individual to be active early or late (12, 13). It is typically assayed by questionnaire, for example, the Horne–Ostberg Morningness–Eveningness Questionnaire [MEQ, (14)], which quantifies subjective time-of-day preference, or the Munich ChronoType Questionnaire [MCTQ, (15)], which measures sleep timing during workdays and free days. MCTQ analysis of a large cohort of subjects in different geographical areas suggests a near-Gaussian distribution for MCTQ coefficient that ranges from extremely early-active to extremely late-active individuals. Such extreme lark and owl chronotypes are barely overlapping in their activity phases (13, 15). Moreover, individual chronotype is evolving during one’s lifetime. Circadian organization of sleep/wake cycles and physiology appears in newborns during the first several months. Its progression is characterized by earliness during childhood moving to lateness that reaches a maximum around the age of 20. Then, a gradual return to earliness is observed with increasing age. Women reach their maximal lateness before men and are then exhibiting generally earlier chronotypes than men, although this sex difference disappears around the age of 50 (13, 16). Social behavior impacts on both chronotype and sleep duration. Indeed, sleep duration and timing are often different during work and free days, and depend on sleep debt accumulation during the week, as well as on social interactions (15). Timing of the sleep/wake cycle is a complex trait that involves many genes and their interactions with environmental factors. Genetic linkage and association studies have resulted in the identification of genetic variants associated with period length and entrainment of the circadian clock (17–22).

Modern lifestyle can be associated with prolonged exposure to artificial light, late meal times, sleep curtailment, potentially rotation shift work into the night-time, and frequent intercontinental time-zone changes. These common aspects of the industrialized world can generate a misalignment between internal circadian clocks and the external light–dark cycle. Such a situation is exacerbated by the frequent difference in the timing of sleep on workdays and free days, leading to a chronic circadian phaseshift known as “social jetlag” (9, 23, 24). Perturbation of circadian rhythms in animals and humans through simulated or actual shift work has been well-documented to interfere with numerous aspects of health (25), and to provoke pathological conditions, including metabolic diseases such as obesity and type 2 diabetes (T2D), cardiovascular diseases, thrombosis, or cancer (9, 10, 26–32). Moreover, chronic sleep and circadian disruption caused extensive inflammation (33), modulated cortisol levels and significantly increased C-reactive protein (CRP), tumor necrosis factor α (TNFα), and other inflammatory cytokine levels in plasma (34). Of note, the occurrence of social jetlag can be linked to individual chronotype. One striking example is that of teenagers exhibiting later chronotype than younger children and adults, and therefore often suffering from chronic social jetlag due to the obligation to cope with early school opening hours. Moreover, individuals with extreme early and extreme late chronotypes might exhibit vastly different reactions to the same shift work schedule, depending on their natural morningness or eveningness (35). Due to accumulating evidence about the detrimental effects of chronic circadian misalignment upon quality of life, professional performance, and health, it becomes evident that individual chronotype should be regarded as an important parameter for one’s rest-activity routine schedule.

## Human Peripheral Clocks: An Important Diagnostic Tool and Therapeutic Target

### Experimental approaches for studying human clocks *in vivo*

The elucidation of the emerging link between clock alterations, individual chronotype, and disease necessitates a molecular dissection of individual clock properties in physiological conditions, and as a result of circadian misalignments. This requires prolonged and regular subject observation and sampling procedures under controlled laboratory conditions. Current protocols are typically based upon “forced desynchrony” or “constant routine” procedures in which individuals deliberately maintain non-circadian schedules under carefully controlled conditions, while monitoring the timing of the circadian hormone melatonin and/or circadian variations in body temperature (36, 37). Although such studies remain the “gold standard” for the determination of human behavioral period length, they are expensive and labor-intensive, and require considerable subject commitment.

Less elaborate methods for studying human clocks *in vivo* have been developed to study individuals in home environments [reviewed in Ref. (38)]. Among relatively non-invasive methods, continuous recording of thoracic skin surface temperature (39, 40) or periodic recording of urinary or salivary melatonin (41) can yield biological circadian phase information if not free-running circadian period. A second type of measurement developed in the past decade relies upon serial sampling of biological matrices such as oral mucosa biopsy (42), hair follicle (43), suction blister content (44), blood, and saliva. For example, by collecting saliva and blood samples “around-the-clock”, diurnal changes in the levels of plasma melatonin (45), cortisol (46), thyroid hormones, insulin, and many other hormones and cytokines can be assessed (47, 48). In a more elaborate approach, timing and amplitude of internal body rhythms have been assessed by large-scale circadian metabolome and transcriptome analysis in blood samples (49–51). Moreover, metabolome analysis of saliva samples, collected in a circadian manner, provided interesting clues to free fatty acids, amino acids, and other metabolites exhibiting strongly oscillatory profiles (52). Remarkably, non-invasive large-scale real-time breath metabolome analysis, or “breathprinting,” has been recently proposed (53), significantly enhancing the speed and ease of sample collection.

However, marker-based methods often suffer from the relative variability of the markers employed. Melatonin, although the standard reference for precise timing of circadian phase, provides no measure of circadian amplitude because of variations in pineal size and calcification (54, 55), and requires numerous serial measurements. Although transcriptomic and metabolomic methods could in principle use many markers of different phase to estimate timing with only a single timepoint, inter-individual variability in marker expression has greatly limited the precision of these techniques so far (50, 53). Collectively, these methods represent a significant step forward and brought important new insights into the human circadian clock.

### *In vitro* synchronized human primary skin fibroblasts as a powerful tool for studying human circadian oscillators

In view of the difficulty of methods for *in vivo* clock studies in humans, extensive efforts have been undertaken aiming at establishing novel approaches for assessing inter-individual differences in circadian amplitude, phase, and free-running period using *in vitro* cultured human primary explants/cells. Experiments performed in immortalized mouse and rat fibroblasts revealed that circadian clocks can be synchronized *in vitro* by multiple signaling pathways allowing the subsequent measure of circadian gene expression for several days (56–59). Fluorescent and bioluminescent circadian reporters represented an additional important breakthrough in circadian clock studies. Among other important information provided by this methodology, it allowed for elegant and direct demonstration of peripheral clocks as cell-autonomous (60).

**Figure 1:**
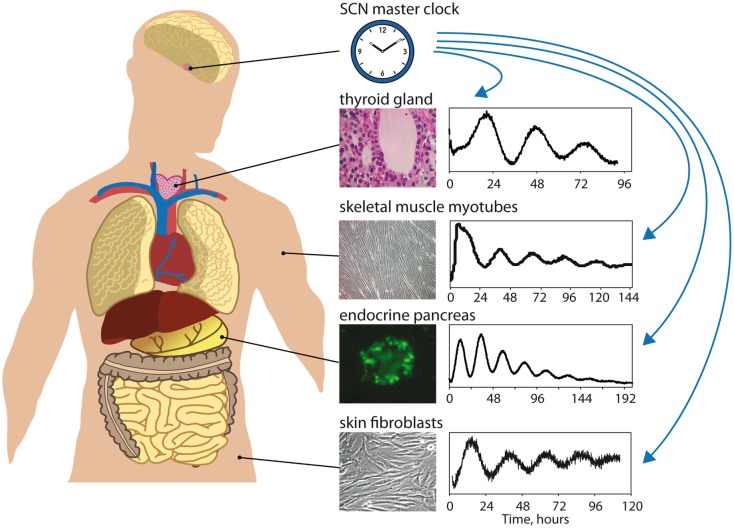
**Studying peripheral oscillators in humans [adapted from Ref. (10) with permission]**. The master clock in the suprachiasmatic nuclei (SCN) of the hypothalamus maintains phase coherence between peripheral oscillators present in virtually all cells of the body by means of daily synchronizing cues (hormonal signals, neuronal signals, rest/activity and feeding/fasting control, body temperature regulation). Circadian gene expression of different peripheral tissues, such as thyroid gland (68), skeletal muscle myotubes (Laurent Perrin and Charna Dibner, unpublished), pancreatic islets (69), or skin fibroblasts (61, 63), can be monitored *in vitro* in synchronized cultured cells from patients biopsies or donors samples using bioluminescent circadian reporters (*Bmal1-luciferase* in this scheme). Circadian properties of these oscillators (phase, period, amplitude, magnitude, resetting) can be analyzed to give subject-specific circadian phenotype information that might be included in diagnostic procedures in the near future.

Our recent studies, employing these important experimental advances, provided convincing evidence that cultured primary human skin fibroblasts expressing circadian bioluminescence reporters represent an excellent experimental system for the dissection of oscillator properties [Figure 1; (61)]. In addition, the same cells could in the future provide substrates for biochemical or genetic analysis of the mechanisms underlying these properties. Of note, circadian clock parameters measured by the continuous recording of circadian bioluminescence cycles produced by human skin fibroblasts vary widely among the cells harvested from different donors (61, 62). Importantly, circadian oscillator characteristics measured in cultured skin fibroblasts correlate with rhythmic human behavior, as evaluated on the basis of human subjects whose circadian physiology was examined under laboratory conditions (63) or individuals completing a questionnaire (62, 64–66). These studies demonstrated that long and short periods in fibroblast clock gene expression were associated with long (“owl-like”) and short (“lark-like”) chronotypes, respectively (62). Moreover, the effect of human blood-borne factors on the period length of circadian gene expression in cultured human fibroblasts cells has been studied in this system. Remarkably, cultured fibroblasts exposed to serum collected from elderly people displayed shorter periods than fibroblasts exposed to serum harvested from young individuals (67). This suggests that circadian molecular oscillators are plastic: they can change their properties according to their environment.

### Human circadian clock properties as a hallmark of the disease

**Figure 2:**
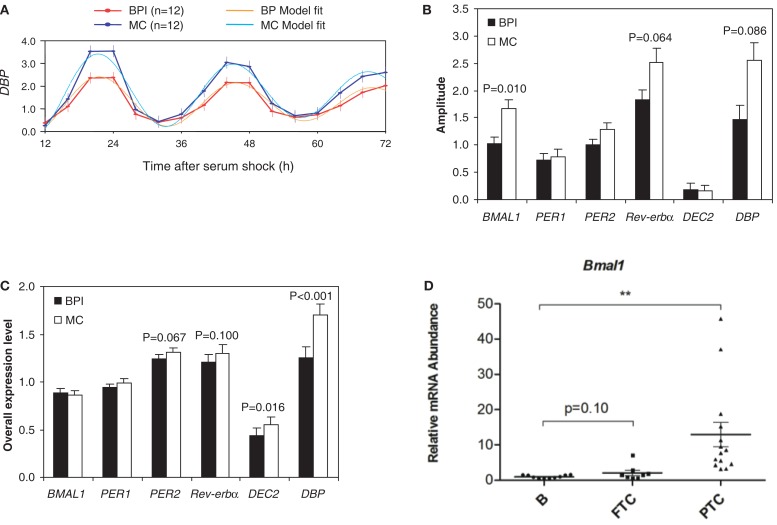
**Disease-associated alterations of circadian function in human patients**. **(A)** The amplitude of expression of *Dbp* after serum shock is reduced by 35% in fibroblasts from bipolar patients (BPI) as compared to healthy matched controls (MCs). Amplitude of rhythmic gene expression, defined at 12 h after serum shock for a series of individual gene **(B)** or overall relative expression levels of each individual genes **(C)** in fibroblasts from bipolar patients and age- and gender-matched unrelated controls. **(D)** Alterations of the expression of *Bmal1* core-clock gene in tissue biopsies of follicular thyroid carcinoma and papillary thyroid carcinoma as compared to benign thyroid nodules (**P < 0.01) **(A–C)** were adapted from Ref. (73), and **(D)** was adapted from Ref. (68), with permissions.

In view of the correlation between oscillator properties assessed *in vitro* in human skin fibroblast experiments and circadian phenotype observed *in vivo*, one important application of fibroblast-based methodologies would be to examine changes in clock properties in different pathological conditions. For example, we have already discussed that some age-related circadian changes are reflected in human skin fibroblasts cultured in the presence of serum from aged subjects (67, 70). In line with these findings, disturbed circadian behavior in bipolar disorder (71, 72) was reflected in reduced amplitude of clock gene expression in fibroblasts [Figures 2A–C; (73)] in one study, and changes in the amplitude of the clock-associated CREB signaling in another (74). If similarly dysregulated in the brain, such circadian changes may contribute to the etiology of depressive disorders, or alternatively be the consequence of disorder-related changes.

Extension of such approaches to primary cell culture established from various peripheral organs holds further promise to obtain tissue-specific information on the molecular makeup of human clocks and their roles in numerous aspects of physiology and pathophysiology. Indeed, robust circadian oscillations have been observed in human pancreatic islets kept in organotypic cultures or as dispersed islet cells [Figure 1; (69)], and in primary human skeletal myotubes differentiated *in vitro* (Figure 1, Laurent Perrin and Charna Dibner, unpublished data). Recent evidence suggests a link between circadian clock perturbations and metabolic diseases in humans (see above), and work in rodents shows an essential role of the circadian clock in insulin secretion by the pancreatic islet (75), as well as in insulino-sensitivity by the skeletal muscle (76). Therefore, studying the properties of human pancreatic islet and skeletal muscle clocks in primary culture may represent an important and unique approach for understanding the etiology of obesity and T2D, and potential connections of the clock to metabolic diseases. For example, the levels of mRNAs encoding PER2, PER3, and CRY2 proteins are decreased in pancreatic islets isolated from T2D donors in comparison to their healthy counterparts (77). Characterization of circadian profiles of synchronized healthy vs. T2D human islets might provide information about the mechanism underlying these and other changes in clock function as a result of this disease, and how they relate to transcriptomic and functional changes within the pancreatic islet.

Similarly, circadian bioluminescence patterns of skeletal myotubes from obese subjects revealed a tendency toward reduced circadian amplitude in these individuals (Laurent Perrin and Charna Dibner, unpublished), in agreement with findings in rodents (78). Robust circadian reporter oscillations have also been recorded in human primary thyrocytes established from thyroid biopsies [Figure 1; (68)]. In contrast, synchronization properties of thyrocytes were altered in cells established from papillary thyroid carcinoma [PTC; (68)]. Moreover, strong alterations of BMAL1 and CRY2 expression levels in PTC thyroid nodule tissue biopsies were observed in comparisons to benign counterparts in multiple studies [Figure 2D; (68, 79)]. In view of the strong emerging connection between cellular circadian clock alterations, malignant transformation, and its outcome [(68, 80–83); reviewed in Ref. (84–86)], these differences in clock function between malignant and benign nodules could hold potentially important implications for preoperative thyroid cancer diagnostics.

The examples cited above directly underscore the potential relevance of circadian studies in primary human cells for metabolic diseases and for cancer. Certainly, such *in vitro* experiments do not necessarily recapitulate the multifactorial aspect of the *in vivo* environment. However, this simplification can be considered as an advantage, since it allows dissection of oscillator function in the absence of interference by physiological state (light input, sleep-wake cycle feedback, blood-born factors), and thus reflects inherent characteristics of individual molecular clocks and potential durable alterations of this clockwork in pathological situations. Not only genetic alterations, but also epigenetic ones due to either disease or its treatment could be potentially studied *in vitro* in this way.

Such isolation can be particularly useful for examining effects of pharmacological treatments specifically upon the circadian system. For example, in bipolar disorder, not only has it been shown that pharmacological treatment of human fibroblasts by valproic acid or lithium can alter circadian clock properties (87–89), but also in reverse that clinical pathology can predict their effects upon the clockwork (90). Thus, we propose that peripheral tissue circadian diagnostics could hold implications for personalized pharmacotherapy in a wide variety of disorders.

In a related fashion, circadian drug delivery already plays an important role for some common drugs like statins and holds considerable promise for oncology, metabolic, and respiratory disease. Therefore, determination of circadian body timing could be key to efficacy (86, 91). While chronic measures in primary culture lose this timing information, acute sampling methods could provide it, adding an additional reason to collect and analyze such samples for a wide range of pathologies.

## Conclusion and Perspectives

Recent studies have demonstrated that cultured primary human skin fibroblasts represent a non-invasive and informative experimental system allowing assessment of circadian clock function in humans (62). Moreover, extending this approach to human primary cells established from different organ biopsies has offered the opportunity to gain important molecular insights into human peripheral clockwork (68, 69). Continuous monitoring of circadian gene expression in cultured human primary cells synchronized *in vitro* may not only allow the characterization of individual circadian phenotype, but also bring new insights into the connection between circadian oscillator function and the etiology of metabolic disorders or cancer. This approach may therefore give important information for personalized medicine. If specific alterations of circadian function are associated with a disease, one might imagine oscillator modulation as a new therapeutic approach for management of this disease (92, 93), or as markers for its diagnosis (68, 79). Given the increasingly apparent importance of circadian clock function in homeostasis and metabolism, the knowledge of individual circadian phenotype may become meaningful in a broader range of circumstances than expected, and have immediate clinical implications.

## Conflict of Interest Statement

The authors declare that the research was conducted in the absence of any commercial or financial relationships that could be construed as a potential conflict of interest.
